# Exploring biorefinery alternatives for biowaste valorization: a techno-economic assessment of enzymatic hydrolysis coupled with anaerobic digestion or solid-state fermentation for high-value bioproducts

**DOI:** 10.1080/21655979.2024.2307668

**Published:** 2024-01-24

**Authors:** Esther Molina-Peñate, Adriana Artola, Antoni Sánchez

**Affiliations:** GICOM Research Group, Department of Chemical, Biological and Environmental Engineering, School of Engineering, Edifici Q, Universitat Autònoma de Barcelona, Barcelona, Bellaterra, Spain

**Keywords:** Techno-economical assessment, biorefinery, organic fraction of municipal solid waste, valorization, enzymatic hydrolysis, anaerobic digestion, solid-state fermentation, biopesticide, sensitivity analysis

## Abstract

Enzymatic hydrolysis of organic waste is gaining relevance as a complementary technology to conventional biological treatments. Moreover, biorefineries are emerging as a sustainable scenario to integrate waste valorization and high-value bioproducts production. However, their application on municipal solid waste is still limited. This study systematically evaluates the techno-economic feasibility of the conversion of the organic fraction of municipal solid waste (OFMSW) into high-value bioproducts through enzymatic hydrolysis. Two key variables are examined: (a) the source of the enzymes: commercial or on-site produced using OFMSW, and (b) the treatment of the solid hydrolyzate fraction: solid-state fermentation (SSF) for the production of biopesticides or anaerobic digestion for the production of energy. As a result, four different biorefinery scenarios are generated and compared in terms of profitability. Results showed that the most profitable scenario was to produce enzymes on-site and valorize the solid fraction via SSF, with an internal rate of return of 13%. This scenario led to higher profit margins (74%) and a reduced payback time (6 years), in contrast with commercial enzymes that led to an unprofitable biorefinery. Also, the simultaneous production of higher-value bioproducts and energy reduced the economic dependence of OFMSW treatment on policy instruments while remaining energetically self-sufficient. The profitability of the biorefinery scenarios evaluated was heavily dependent on the enzyme price and the efficiency of the anaerobic digestion process, highlighting the importance of cost-efficient enzyme production alternatives and high-quality OFMSW. This paper contributes to understanding the potential role of enzymes in future OFMSW biorefineries and offers economical insights on different configurations.

## RESEARCH HIGHLIGHTS

- Techno-economic analysis to assess enzyme origin and solid hydrolysate fate.
- The viability of enzymatic hydrolysis depends on the cost and origin of enzymes.
- On-site produced enzymes cut payback time to 6 years, elevating profits by 74%.
- Anaerobic digestion and solid-state fermentation can be complementary technologies.
- High-value bioproducts are key to making organic waste biorefineries profitable.

## 1. Introduction

In recent years, the growing environmental concern and the energy crisis have accelerated the development of innovative waste management and treatment technologies. Particularly, the increasing generation of municipal solid waste (MSW), primarily composed of organic waste, has prompted society to seek resources and energy recovery from waste [1]. The organic fraction of municipal solid waste (OFMSW), commonly referred to as biowaste, is composed of food residues and green waste, and it is suitable for valorization through biological treatments [2].

As separate collection systems for OFMSW expand, both the quality of OFMSW and its collection costs increase [3,4]. Therefore, it is becoming increasingly apparent that waste valorization alternatives beyond well-established and robust methods, such as composting and anaerobic digestion, are necessary to maximize profitability. In this regard, biorefinery-like configurations appear as an alternative to the traditional OFMSW treatment plants [5]. Biorefineries are sustainable bioprocessing facilities that optimize revenue generation from the original feedstock while also reducing impacts on natural resource consumption [6]. This is achieved by integrating different conversion methods to produce multiple marketable bioproducts [1]. According to the cascading principle, added-value products should be produced first followed by energy generation [7]. Currently, the preferred configuration for source-selected OFMSW treatment plants in Europe is anaerobic digestion for biogas production, which is used to generate electricity and heat, followed by composting to stabilize the digestate and produce compost [8]. However, within an OFMSW biorefinery scheme, processes that fractionate or convert complex organic matter into a wide variety of bioproducts would come before anaerobic digestion. By doing so, biorefineries improve the sustainability of waste management in line with EU circular economy policies [9].

Several biorefinery configurations have been proposed to convert the OFMSW into value-added products such as biosurfactants, sugar syrups, bioethanol, succinic acid, lactic acid and biopesticides [10–15] and in-depth reviewed elsewhere [4,5]. A common trait among many of these is the use of enzymatic hydrolysis to fractionate the complex OFMSW macromolecules into functionalized molecules, which act as building blocks for subsequent steps [5,16]. Comprising 45–85% of the OFMSW composition [16], carbohydrates and fibers serve as a source of fermentable sugars, capable of being converted into bio-based products via fermentation processes [13]. However, these studies are mostly focused on the liquid fraction rich in sugars and either disregard the solid fraction or consider it a waste and direct it to anaerobic digestion. Therefore, further research is needed to integrate the solid hydrolyzate fraction into the overall valorization pathway of biorefineries.

Our recent study demonstrated the utilization of the solid fraction remaining after enzymatic hydrolysis for *Bacillus thuringiensis* (Bt) biopesticide production through solid-state fermentation (SSF) in a 22 L bench-scale bioreactor [17]. A final concentration of 4 × 10^8^ spores of Bt per gram of dry matter was obtained by mixing the solid hydrolyzate with solid digestate, which has been also demonstrated as a suitable substrate for biopesticides production through SSF [18,19]. SSF technology is attracting increasing interest due to its low water and energy requirements without compromising yield [20]. Biopesticides are biological agents that offer a promising alternative to chemical pesticides for the control of pests in agriculture [21]. The global market for biopesticides is growing at an annual rate of 15% [22], driven by rising awareness of the environmental and health risks of chemical pesticides, increased demand for organic and sustainable agricultural products, and government regulations that favor the use of biopesticides [21]. This market is dominated by biopesticides derived from *Bacillus thuringiensis*, which represent over 80% of the global biopesticide market [23].

OFMSW stands out as a unique feedstock given its inherent variability, heterogeneity, complex structure, and indigenous microbial consortium. The robustness and efficiency of anaerobic digestion technology for the conversion of OFMSW into biogas and biomethane has positioned it as an essential foundation upon which to build an OFMSW biorefinery [24]. Furthermore, biogas can be converted into electricity and heat or upgraded to be pumped into the natural gas grid, converting it into an economically compelling choice. Therefore, novel integrated OFMSW biorefineries should built on existing treatment facilities using anaerobic digestion.

The OFMSW biorefinery configuration proposed in this study is based on the use of enzymatic hydrolysis to obtain a sugar syrup and SSF to convert the subsequent solid hydrolyzate together with solid digestate into a solid-state biopesticide. However, it is well known that the main obstacle in implementing enzymatic hydrolysis at an industrial scale is the high cost of commercial enzymes [25], considering that the complex nature of OFMSW hampers enzyme immobilization or recovery. To avoid the market cost of enzymes, their production could be integrated into the biorefinery. Fungal species have been widely exploited in SSF processes due to their enzyme battery and their ability to grow on solid substrates [20]. Table 1 summarizes different types of enzymatic activities that have been produced by SSF using organic wastes. The inoculum employed mainly belongs to the fungal genus of *Aspergillus* or autochthonous microbiota of waste materials. Specifically using OFMSW as substrate, Ladakis et al. [13] proposed on-site production of crude enzymes within an OFMSW biorefinery dedicated to succinic acid production. This approach involved less than 20% of the total capital investment and reduced processing costs. However, it is important to evaluate whether the reduced operating cost compensates for the additional capital investment required. Another major economic advantage of producing enzymes on-site is the reduced formulation requirements as long-term storage and transportation are avoided and the formulation can be adjusted to the immediate application, reducing downstream purification steps required in more complex processes [5,13].

**Table 1.** Examples of published works on SSF for enzyme production.

Enzymes	Inoculum	Substrate	SSF scale	Solid load	Air flow	SSF Temperature	SSF time	Enzyme yield	Reference
Pectinases	*Aspergillus oryzae*	Citrus waste & sugarcane bagasse	400 L	15 kg	0.15 L h^−1^	28–30°C	24 h	37 U g^−1^	[26]
Cellulases and xylanases	Specialized consortium from compost	Coffee husk	50 L	15 kg	NM	25–60°C (NC)	24 h	47 U g^−1^ DM	[27]
Cellulases and xylanases	*Aspergillus niger*	Brewer’s spent grain	0.5 L	0.1 kg	0.05 L h^−1^	30°C	96 h	268 U g^−1^ DM	[28]
Amylases	*Bacillus sp.*	Agroindustrial waste	0.25 L	0.05 kg	-	40°C	48	82 U g^−1^ DM	[29]
Cellulases, proteases and glucoamylases	*Aspergillus awamori*	OFMSW	0.25 L	0.05 kg	-	30°C	96 h	36 U g^−1^ DM	[13]

NM, not mentioned. NC, not controlled. DM, dry matter.

In this study, the economic performance of an OFMSW biorefinery integrating enzymatic hydrolysis into an anaerobic digestion treatment plant is explored. Process design, techno-economic assessment and investment profitability have been systematically studied to show the potential of enzymatic hydrolysis for the treatment of OFMSW. Four scenarios are proposed to evaluate the allocation of the solid hydrolyzate for biopesticide production by SSF or for energy production by anaerobic digestion, as well as to study the origin of the enzymes, whether they are commercially sourced or produced on-site. The aim is to compare these scenarios and identify the biorefinery configuration that is most profitable for potential implementation. Currently, this is one of the few articles evaluating in detail the role of enzymatic hydrolysis from a techno-economic perspective in future OFMSW biorefineries.

## 2. Materials and methods

### 2.1. Simulation description

The proposed biorefinery was simulated with a processing capacity of 300 t per day of source-separated OFMSW, which has been assumed to contain a high organic matter percentage and be free of inert materials. The simulation begins at the gate of the biorefinery, so the cost of waste collection or transportation has not been considered. The plant has been located in Barcelona (Spain) with a 20-year lifetime, including three years of construction and the start-up phase. It operates 333 days per year, corresponding to an annual processing capacity of 100,000 tonnes, which is equivalent to a population of approximately 507,000 inhabitants based on the average waste generation rate in Catalonia [30]. Mass and energy balances of the processes and all economic calculations have been conducted with Microsoft® Office Excel (version 2019).

### 2.2. Process description

**Figure 1.**
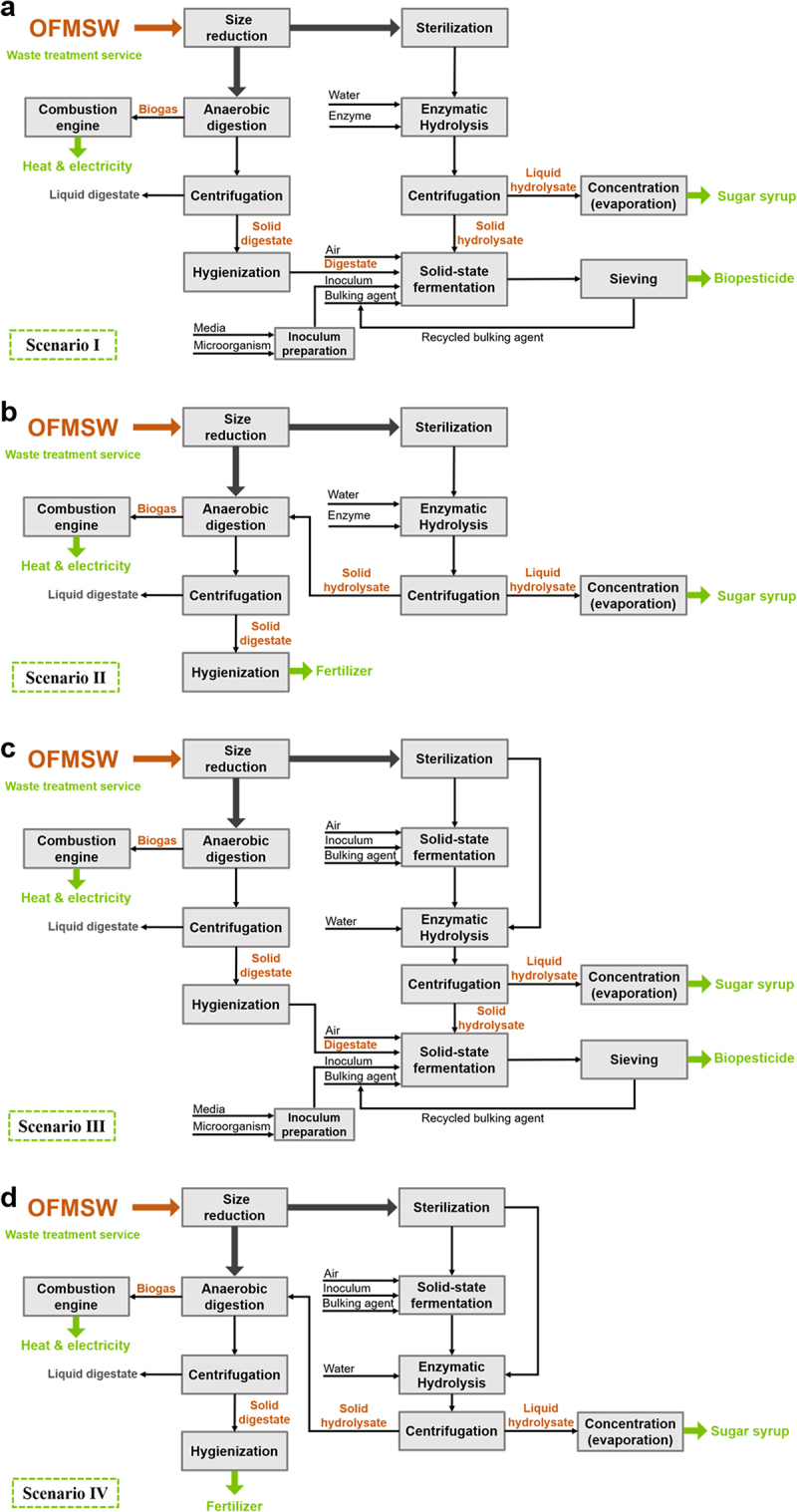
Process flowchart of the different biorefinery scenarios for the application of enzymatic hydrolysis in OFMSW treatment. (a) Scenario I, commercial enzymes and solid hydrolysate valorization through SSF. (b) Scenario II, commercial enzymes and solid hydrolysate valorization through anaerobic digestion. (c) Scenario III, *in situ* produced enzymes and solid hydrolysate valorization through SSF. (d) Scenario IV, *in situ* produced enzymes and solid hydrolyzate valorization through anaerobic digestion.

The main bioconversion process presented in this study has been previously demonstrated at laboratory and bench scales, providing the technical data needed to perform the simulation [17,31]. When required, the experimental data was complemented with data from literature, as indicated. The biorefinery configurations proposed are based on three main technologies, namely enzymatic hydrolysis, solid-state fermentation and anaerobic digestion. Depending on the allocation of the solid enzymatic hydrolyzate and the origin of the enzymes, four scenarios are proposed to investigate alternatives for implementing enzymatic hydrolysis in the treatment of OFMSW. As shown in Figure 1, Scenarios I and II evaluate the use of commercial enzymes, with Scenario I using the solid hydrolyzate in SSF for biopesticide production and Scenario II in anaerobic digestion for energy production. Meanwhile, scenarios III and IV explore the use of on-site produced enzymes with analogous solid hydrolyzate allocation strategies. The main parameters used for developing the process simulations are summarized in the supplementary material (Table S1).

#### 2.2.1. Pretreatment area

The process begins with a primary shredder to reduce the size and homogenize the OFMSW. Then, the shredded OFMSW is split into 70% into the current energy valorization line via anaerobic digestion and 30% into the novel enzymatic hydrolysis valorization line. This ratio remains the same for all four scenarios. For the enzymatic hydrolysis route, an autoclaving step has been introduced to minimize the microbial load. It also serves as a mild hydrothermal pretreatment for lignocellulosic materials, increasing cellulose accessibility to enzymes while generating fewer inhibitory compounds than harsher pretreatment methods [12]. A 20 t batch size has been assumed based on a previous study, in which the authors used a pilot scale autoclaving system for MSW that required 12 kWh of electricity, 76 kWh of natural gas and 245 L of water per ton of waste [12].

#### 2.2.2. Enzymatic hydrolysis

The enzymatic hydrolysis to extract sugars from the OFMSW is performed in a closed tank at a 10% solid-to-liquid ratio (w/v), which is adjusted by adding distilled water and considering that the moisture content in the autoclaved OFMSW is 75% [31,32]. The commercial enzymatic cocktail selected is Viscozyme L® and it is dosed at 0.08 mL g^−1^ of dry OFMSW, as optimized in previous works [31]. The mixture is then heated to 50°C for 24 h. Two hydrolysis tanks operate in batch mode, considering a total operation time of 48 h to account for the time for loading and unloading the tanks. After hydrolysis, a concentration of 50 g L^−1^ of reducing sugars is achieved in the liquid fraction [31,33]. This liquid fraction is recovered by a decanter centrifuge assuming that it accounts for 74% of the initial fresh substrate mixture [17]. Finally, water is evaporated in a drying step until achieving a final concentration of 50%, which is acceptable for mixed sugar syrups from lignocellulosic materials [34]. The solid hydrolyzate fraction recovered in the centrifuge is used as a substrate for SSF (Scenarios I and III) or, alternatively, for anaerobic digestion (Scenarios II and IV) (Figure 1). To simulate the energy demand for heating the mixture to the hydrolysis temperature, a heat capacity of 3.3 kJ kg^−1^ K^−1^ was calculated using the correlation presented by Manjunatha et al. [35] for MSW considering a moisture content of 75%. The concentration step is performed by a flash bed dryer with an energy consumption of 3 MJ kg^−1^ of evaporated water [36].

#### 2.2.3. Solid-state fermentation for biopesticide production

In Scenarios I and III (Figure 1) the solid hydrolyzate obtained after the enzymatic hydrolysis is used to produce a fermented solid with biopesticide activity through SSF. In an SSF bioreactor, the solid hydrolyzate is mixed with pasteurized solid digestate and wood chips that act as bulking agent, in a 1:1:0.5 ratio (w/w) [17]. Then, the substrate mixture is inoculated with *Bacillus thuringiensis* var *israelensis*, which has been previously grown in liquid media in a seed bioreactor. To reach an initial concentration of 10^7^ viable cells g^−1^ of dry matter, around 25 L of inoculum per ton of substrates are required assuming an inoculum concentration of 10^8^ viable cells mL^−1^ [17]. The process begins when the mixture is forcefully aerated with 250 m^3^ of compressed air per ton per day and lasts for six days, considering one day for loading, unloading and cleaning the bioreactor [17]. After 5 days the fermented solid is sieved to recover the bulking agent, assuming a 90% recovery efficiency (personal communication with waste treatment plant manager). To simulate the energy consumption for the SSF process, it has been assumed that no heating is required, as heat accumulates in the solid matrix during the fermentation course [17]. Indeed, temperature is controlled by introducing cold air. Using a conversion factor of 396 KJ *m*-^3^, the air supply is transformed into the total energy consumed, resulting in a specific energy consumption of 27.5 kWh t^−1^ of substrate [37]. For the inoculum preparation, a power consumption of 4 kW m^−3^ is considered [38]. Lastly, for the sieve, an electric power of 40 kW is assumed (Terra select T4 ®).

#### 2.2.4. Anaerobic digestion

The majority (70%) of the incoming OFMSW into the plant is processed through anaerobic digestion to produce energy, as well as the solid hydrolyzate produced after enzymatic hydrolysis in Scenarios II and IV (Figure 1). For the simulation of the anaerobic digestion process, data from a local municipal OFMSW treatment plant has been used [39]. The grinded OFMSW, which has a dry matter content of 25% [17], is mixed with water until a 15% dry matter content is achieved. Mesophilic anaerobic digestors of 3000 m^3^, with a hydraulic retention time of 16 days and a loading rate of 3.0 kg VS m^−3^ day^−1^, have been assumed. A biogas production rate of 151 m^3^ t^−1^ OFMSW, with a 64% methane content is considered based on a previous work using a high-quality source separated OFMSW [40]. The obtained biogas is used to produce heat and power by means of a biogas engine (CHP unit) [39]. According to Tampio et al. [41], the quantity of produced digestate is calculated by subtracting the mass of biogas from the total input material (including water) and taking into account the biogas composition and the component densities (CH_4_ 0.72 kg m^−3^ and CH_2_ 1.96 kg m^−3^). Another decanter centrifuge is used to recover the digested solids considering a rate of 20% of the input [41], which then is transferred to a hygienization unit for pasteurization, as specified in European Regulation N◦ 142/2011, before being used as a cosubstrate in the SSF in Scenarios I and III (Section 2.2.3) or as fertilizer in Scenarios II and IV (Figure 1). In this study, the fate of the liquid digestate has not been considered as the scope is within the solids fraction and previous works have extensively evaluated it [40,41]. To simulate the energy consumption of the anaerobic digestion process, an average electric energy consumption of 0.2 kWh t^−1^ of input material [41,42] has been considered. Thermal energy consumption has been specifically calculated as the energy required to heat the input material (grinded OFMSW and water) until mesophilic temperatures (40°C). To do so, it is assumed that the specific heat capacity of the input flow is the same as water (4.18 kJ kg^−1^ ºC^−1^) as in [41]. The energy required for heating the solid digestate in the hygienization step (75°C) is calculated following the same principle (see Supplementary material).

The conversion of the produced biogas into heat and electricity in the CHP unit was done by calculating the electricity yield (kWh t^−1^) using the methane content (64%), the conversion factor of 10 kWh per m^3^ of methane and the conversion efficiencies in the CHP unit of 38% for electricity and 48% for heat [41,43,44]. The energy consumption of the CHP unit is assumed to be 10% of the total energy produced [41]. The generated heat is assumed to be used in the plant using a heat exchanger system for heating applications (anaerobic digestion, hygienization, enzymatic hydrolysis and drying steps) [39]. A 20% of the total heat produced has been accounted for as heat losses [45]. Electricity is also assumed to cover the plant requirements and, when excess is produced, to be sold to the electrical grid.

#### 2.2.5. Solid-state fermentation for enzyme production

In Scenarios III and IV (Figure 1) it is proposed that the enzymes consumed in the enzymatic hydrolysis are produced on-site since it is well-known that the cost of commercial enzymes is a limiting factor for their application [25,46]. To do so, an SSF process for enzyme production has been simulated based on data from previous studies [13,47]. It has been demonstrated that OFMSW is an adequate substrate to produce crude enzymes using fungal species, such as *Aspergillus awamori* [13,48]. For the simulation, 50% of the grinded and autoclaved OFMSW has been mixed with a bulking agent and inoculated with *A. awamori* at a concentration of 10^6^ spores g^−1^, using an inoculum previously grown in seed bioreactors. The SSF process duration is five days including a day for loading and unloading operations. This fermented solid rich in crude enzymes was mixed with the remaining 50% of the sterile OFMSW, diluted with distilled water to attain a solid load of 20% and heated to 50°C following the conditions explained in Section 2.2.2. According to [13], the fermented solid contained 24 U g^−1^ of maltase activity, 39 U g^−1^ of glucoamylase activity and 2 U g^−1^ of cellulase activity, whereas the reported activity for Viscozyme L® is ≥ 100 FBU g^−1^. It was decided to use the whole SSF solids to ensure a complete utilization of the sugars in the OFMSW and simplify the process.

### 2.3. Economic analysis

To compare the different scenarios, their economic performance was studied by estimating the capital cost, operation cost and revenue generation. Then, cumulative cash flow was calculated and the profitability was assessed by evaluating different techno-economic indicators.

#### 2.3.1. Total capital cost estimation

The total capital cost includes the fixed capital investment (FCI) and the working capital cost. The FCI refers to the total cost of designing, constructing, installing, and modifying the plant [49]. It is estimated by applying calculation factors to the purchase cost of the equipment for mixed fluids-solids processing plants [49]. In Table 2 the details of the estimation assumptions for the economic evaluation are presented. For the equipment purchase cost, first, the size and capacity characteristics were estimated based on the data from the material balance (Section 2.2), considering the flow rate and the residence time in each unit [50,51]. Then, the cost was calculated using available recent cost data and the factor method [49–51]. The specifications considered for each equipment, as well as, the details for calculating the equipment purchase cost can be found in the Supplementary material (Table S3 and Table S4). Neither equipment for storage nor for solids movement, such as conveyors or trucks, were considered in this estimation. The working capital cost, which represents the capital needed for maintaining plant operations, is recovered at the end of the plant life [49]. Besides, no further capital cost is considered to be recovered after the lifetime of the plant.

**Table 2.** Parameters for the economic evaluation.

Parameter	Estimation assumption
**General considerations**	
Plant location	Province of Barcelona
Plant capacity	100,000 t of source selected OFMSW year^−1^
Plant availability	333 days year^−1^
**Capital cost**	Capital cost (€) = FCI + working capital
Fixed capital cost (FCI)	
• Direct field cost (DFC)	DFC (€) = equipment cost + installation cost
o Installation cost	Equipment cost × 3.2
•Off-site cost	DFC × 0.4
• Engineering cost	(DFC + off-site cost) × 0.25
• Contingency cost	(DFC + off-site cost) × 0.15
Working capital	(DFC + off-site cost) × 0.15
**Annual operating cost**	Operating cost (€ year^−1^) = FOC + VOC
Fixed operating cost (FOC)	
• Labor cost	15.72 € t^−1^ of OFMSW
• Maintenance cost	DFC × 0.06
• Insurance cost	FCI × 0.01
Variable operating cost (VOC)	
• Utilities cost	Electricity: 0.2213 € kWh^−1^ Water: 2.415 € m^−3^ Demineralized water = water × 2
• Raw materials cost	Viscozyme L®: 13.8 € kg^−1^ Rich media: 200 € m^−3^
• Wastewater cost	4.94 € t^−1^ of OFMSW
• Laboratory & analysis cost	0.72 € t^−1^ of OFMSW
**Revenues**	
Treatment fee	34 € t^−1^ of OFMSW
Mixed sugar syrup	365 € t^−1^
Solid biopesticide	400 € t^−1^
Fertilizer (solid digestate)	100 € t^−1^

#### 2.3.2. Operational cost estimation

The annual operating cost includes the fixed operating cost (FOC), which is equal for all scenarios, and the variable operating cost (VOC), which is dependent on the material flow of each scenario [49]. Abad et al. [39] provided data on operational costs per ton of treated waste for a local OFMSW anaerobic digestion treatment plant, which have been used to estimate the cost of labor, wastewater treatment and analysis based on the processing capacity of the biorefinery as indicated in Table 2. The cost of utilities was obtained from the Catalan Institute of Energy and the Catalan Water Agency as the average price for industries of the last two years (2022–2021) [52,53]. For demineralized water, the cost has been estimated to double the price of raw water as indicated by [49]. The commercial enzyme (Novozymes Viscozyme L ®) used in this study has an indicative cost of around 13.8 € kg^−1^ (15 $ kg^−1^) [54]. The cost of the bulking for the SSF processes has been disregarded because a recovery system has been implemented. Other components in the operating cost are summarized in Table 2.

#### 2.3.3. Revenue estimation

In addition to sales of products, the treatment service fee of OFMSW also generates revenues (Table 2). The OFMSW treatment fee was obtained from the Catalan Agency of Waste [55]. For the mixed sugar syrup obtained after the enzymatic hydrolysis, the price was set to the minimum of the raw sugar market 0.37€ kg^−1^ [56], which was half of that for purified sugar syrups [11]. For the solid biopesticide, an estimation was made based on the quantity of active ingredient (spores of *Bacillus thuringiensis* var *israelensis*) in comparison with available products in the market, such as VectoBac WG®. The price of the solid digestate as fertilizer was set as that of compost from OFMSW [39].

#### 2.3.4. Profitability analysis

The techno-economic indicators used to determine the most cost-effective scenario under the prevailing conditions were the gross profit margin, the net production cost, the pay-back time, the return on investment (ROI) and the net present value (NPV) [49]. First, the net cash flow was calculated over the plant’s lifetime by assuming a 3-year construction period, a corporation tax rate of 25% and 10 years for depreciation. The gross profit margin (%) indicates the portion of revenue remaining after subtracting the operating cost and serves as an indicator of the efficiency of the plant. The net production cost (€ t^−1^) is the operating cost per t of treated OFMSW. The pay-back time (years) refers to the time needed to recover the initial investment cost while the ROI (%) refers to the rate of cash return on that investment. The ROI has been calculated over the lifetime of the plant as indicated in Equation 1:

(1) $$\mathrm{ROI}\left(\%\right)=\frac{\mathrm{cummulative}\;\;\mathrm{net}\;\;\mathrm{profit}}{\mathrm{plant}\;\;\mathrm{life}\;\;\times \mathrm{initial}\;\;\mathrm{investment}}\times 100$$

Finally, the NPV was calculated by discounting the future cash flows, including the initial investment, to the present value. A positive NPV indicates that the scenario is profitable for the entire lifetime of the plant and vice versa. It is calculated according to Equation 2:

(2) $$\mathrm{NPV}\left(\text{\textbackslash{}Euro}\right)=\overset{t=20}{\underset{t=0}{\sum }}\frac{CF_{t}}{{\left(1+i\right)}^{t}}$$

where $CF_{t}$ is the net cash flow during the period t (including the initial investment), and i is the interest or discount rate, which was assumed to be 6%. The interest rate at which the NPV is zero is known as the internal rate of return (IRR) and also indicates the efficiency of the investment. The higher the discount rate the longer it takes for the plant to reach a positive NPV [15].

#### 2.3.5. Sensitivity analysis

Sensitivity analysis is a method to examine the effects of uncertainty in model input parameters on the economic viability of the project and to identify the parameters that have the greatest impact on the outcome [49]. By using NPV as the economic indicator, the analysis provides insight into the level of risk associated with making predictions about the future performance of each scenario. The model assumptions independently evaluated at a ±25% variation from reference values were enzyme cost, biopesticide price, direct field cost, biomass production rate and sugar yield. It is represented in a static tornado diagram as the range of the output variation (NPV) for each variable over the specified range. Furthermore, the effect of unexpectedly higher downtime time was evaluated by varying the production rate while keeping capital and fixed operating costs constant [49].

## 3. Results and discussion

### 3.1. Mass balance and bioproducts obtained

The proposed biorefinery in this study processes 100,000 t year^−1^ of OFMSW, with 25% of dry matter content. Different bioproducts are obtained depending on the scenario evaluated (Figure 1): sugar syrup, solid biopesticide (Scenarios I and III), solid fertilizer (Scenarios II and IV) and energy. Table 3 summarizes the overall component balance for each scenario. Enzymatic hydrolysis was the first bioprocess performed in all scenarios to recover 5,614 t year^−1^ sugars for the commercial enzymes (Scenarios I and II) and 2,954 t year^−1^ sugars for the on-site produced enzymes (Scenarios III and IV). This corresponds to a sugar production yield of 0.2 g of sugar per gram of autoclaved OFMSW, considering that 30% (30,000 t year^−1^) and 15% (15,000 t year^−1^) of the total OFMSW input is directed toward enzymatic hydrolysis, respectively. From the residual solids after the enzymatic hydrolysis 42,138 t year^−1^ (Scenario I) and 32,207 t year^−1^ (Scenario III) of solid biopesticide can be produced by SSF. Therefore, by redirecting a part of the incoming high-quality OFMSW, two high-value products can be obtained besides the energy produced in the anaerobic digestion.

**Table 3.** Overall component balance in a year for each scenario.

Item	Scenario I	Scenario II	Scenario III	Scenario IV
**Inputs**				
OFMSW (t year^−1^)	100,000	100,000	100,000	100,000
Enzyme cocktail (t year^−1^)	720	720	0	0
Rich media (m^3^ year^−1^)	1,289	0	1,358	20
Water (m^3^ year^−1^)	49,350	61,185	49,350	55,577
Demineralized water (m^3^ year^−1^)	46,289	45,000	10,764	9,465
Electricity (MWh year^−1^)	10,614	0	0	0
**Outputs**				
Sugar syrup (t year^−1^)	5,614	5,614	2,954	2,954
Biopesticide (t year^−1^)	42,138	0	32,207	0
Fertilizer (t year^−1^)	0	25,551	0	22,890
Electricity (MWh year^−1^)	0	2,305	10,258	17,561

**Figure 2.**
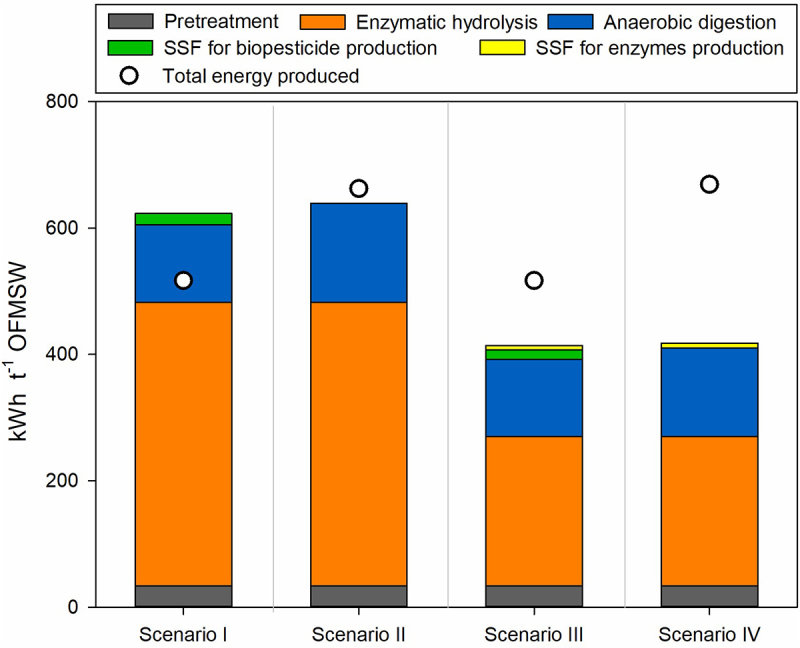
Total energy consumption and production per ton of processed OFMSW in the different biorefinery processes for each scenario.

In scenarios I and III, the solid digestate was used as a cosubstrate in the SSF process, contributing to the control of pH and maintaining its value close to the neutral range, adequate for *Bacillus thuringiensis* growth [17,32]. On the other hand, in scenarios II and IV, where no SSF for biopesticide production is performed, the produced digestate (25,551 t year^−1^ and 22,890 t year^−1^, respectively) is directly sold as fertilizer, which is four times cheaper than the biopesticide (Table 2). In these scenarios, the residual solids after the enzymatic hydrolysis are directed toward anaerobic digestion generating 28% of additional energy. In Figure 2, the total energy consumed and produced in each scenario is presented. The main energy-consuming step in all scenarios is enzymatic hydrolysis, mainly due to the drying equipment for the concentration process (see Supplementary material **Table S3**), which consumes from 68%-66% in Scenarios I and II to 54%-53% in Scenarios III and IV. The decrease in energy consumption in Scenarios III and IV is a result of redirecting a smaller quantity of OFMSW to enzymatic hydrolysis, which consequently leads to a reduced input of liquid hydrolyzate into the concentration step. Water evaporation has been described as a significant energy-consuming step in organic waste biorefineries using enzymatic hydrolysis [57]. Consequently, implementing energy-saving strategies in the drying process can have a significant impact on the overall energy consumption. For example, achieving higher sugar concentrations would result in reduced water evaporation, and thus, leading to lower energy consumption, as assessed later in the sensitivity analysis (Section 3.4). The subsequent energy-consuming step for all scenarios is the anaerobic digestion (Figure 2), mostly due to the heating and operation of the bioreactors and the biogas converting unit (**Table S3**). Scenarios II and IV consume around 4–5% more than Scenarios I and III, respectively, because the solid hydrolyzate is valorized through anaerobic digestion and more bioreactors are required. Consequently, more energy is also produced as observed in Figure 2. In each scenario, the energy consumption of the SSF steps remained below 4%, which might vary if a temperature control system were to be considered. Only Scenario I, which evaluated the use of commercial enzymes and the production of biopesticides from the solid hydrolyzate, exhibited a negative energy balance. Whereas in Scenario II, the additional 146 kWh t^−1^ OFMSW derived from processing the hydrolyzed solid in anaerobic digestion offset the energy requirements, and in Scenarios III and IV, the energy savings in the enzymatic hydrolysis step also lead to positive energy balances.

### 3.2. Capital and operating cost

A summary of the capital and operating costs is presented in Table 4. The capital investment needed for each scenario was calculated based on the cost of the equipment required to perform the processes, as shown in Figure 1, with detailed equipment costs provided in the supplementary material (**Table S4**). The main difference among the four scenarios is that in Scenarios II and IV, the SSF for biopesticide production is not conducted, which accounts for 33% and 26% of equipment cost in Scenarios I and III respectively, hence resulting in capital cost savings. Furthermore, Scenarios III and IV include the additional process of SSF for enzymes production, which increases capital cost by 8% and 10% with respect to Scenarios I and II (Table 4). The main contributor to the capital investment for all scenarios is the anaerobic digestion process, which makes sense considering that it treats 70% of the OFMSW input into the plant, followed by SSF processes. Additional expenses for constructing the plant were projected based on the cost of equipment (**Table S4**).

**Table 4.** Overall economic evaluation of the four OFMSW biorefinery scenarios.

	Scenario I	Scenario II	Scenario III	Scenario IV
**Capital cost**				
Total capital cost (Million €)	68.6	51.0	73.9	56.2
**Operating cost**				
VOC (€ t^−1^ OFMSW)	135	109	9	7
FOC (€ t^−1^ OFMSW)	39	33	41	35
Net production cost (€ t^−1^ OFMSW)	174	142	50	42
**Revenues**				
Treatment fee (€ t^−1^ OFMSW)	34	34	34	34
Sugar syrup (€ t^−1^ OFMSW)	20	20	11	11
Biopesticide (€ t^−1^ OFMSW)	169	0	129	0
Fertilizer (€ t^−1^ OFMSW)	0	26	0	23
Electricity (€ t^−1^ OFMSW)	0	5	23	39
Total income (€ t^−1^ OFMSW)	223	87	196	107
**Profitability analysis**				
Gross profit margin (%)	22	−66	74	60
Pay-back time (years)	15	−10	6.1	10
ROI (%)	5	−10	13	8
NPV (Million €)	−22.6	−97.0	41.2	−2.0

VOC, variable operating cost. FOC, fixed operating cost. ROI, return on investment. NPV, net present value.

Regarding operating costs (Table 4), a noticeable difference can be seen depending on whether the enzymes are purchased (Scenarios I and II) or produced on-site (Scenarios III and IV). This is a result of the high cost of the commercial enzymes, which are responsible for 74% of VOC in Scenario I, where energy costs account for an additional 17%, and 91% of VOC in Scenario II, where energy balance is positive and no input is needed. The sensitivity of the financial analysis to enzyme costs is addressed in Section 3.4. The other contributors to VOC are negligible in comparison (**Table S5**). FOC accounts for 23% of the operating cost in Scenarios I and II and 83% in Scenarios III and IV. Within this category, labor costs remain consistent for all scenarios, while, maintenance and insurance costs are directly related to the capital investment and consequently experience minimal variations. Based on the operating costs, producing enzymes on-site in the biorefinery effectively reduces the net production cost per t of OFMSW treated by half, thereby indicating greater profit margins.

### 3.3. Revenues and investment analysis

The revenues and total income generated per t of OFMSW treated in the different scenarios are shown in Table 4. The profit comes from selling sugar syrup, biopesticides or fertilizer and electricity, and also from the treatment fee, which was equal for all scenarios. This treatment fee represents 14–17% in Scenarios I and III, where the solid biopesticide is produced, and up to 40–32% in Scenarios II and IV, where the solid hydrolyzate is redirected into energy production. Therefore, it is observed that the economic viability of the biorefinery heavily depends on policy instruments when energy production is prioritized instead of high-value products. Scenario I generated the highest revenue at 223 € t^−1^ OFMSW, whereas Scenario II produced the lowest one at 87 € t^−1^ OFMSW. In fact, for Scenario II, the revenues were lower than the net production cost, indicating that the OFMSW was not financially viable in these circumstances. Therefore, the use of enzymes in anaerobic digestion without significant production of higher-value bioproducts is limited by their cost. According to Panigrahi et al. [58], this is the same reason for the limited application of enzymes as a pretreatment of OFMSW. The highest cost allowed to achieve a profitable anaerobic digestion process has been found to be 0.28 € L^−1^ [59], so around 50 times lower than the actual one (Table 2). The market price of novel bioproducts, such as enzymes or solid biopesticides, can depend on different factors, such as intended application or regional policies, so the sensitivity of the economic viability to the selling price of this product is evaluated in Section 3.4.

**Figure 3.**
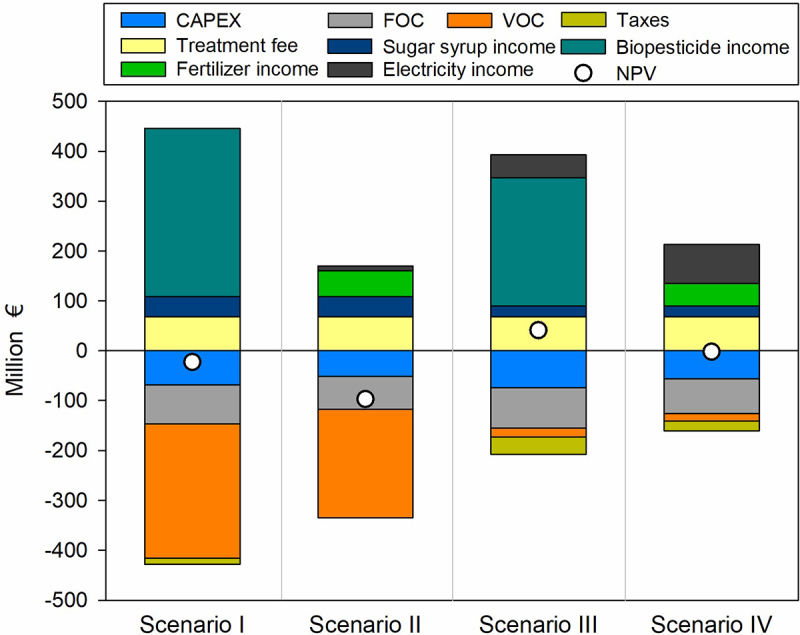
Investment cost, operating cost, revenues and cumulative net present value (NPV) for the four OFMSW biorefinery scenarios. FOC, fixed operating cost. VOC, variable operating cost.

Scenario III, with on-site enzymes production and the use of the solid hydrolyzate for biopesticide production, was the only scenario that proved to be profitable under the circumstances evaluated, as indicated by a positive NPV for the lifetime considered at a 6% discount rate (Figure 3). Taxes are only present in scenarios with a positive net profit. Scenarios I and IV presented a positive gross margin, a payback time lower than the considered lifespan of the plant, and a positive ROI, but a negative NPV. For Scenario I, with commercial enzymes and biopesticide production, it can be observed that the VOC are substantial (Figure 3) and not efficiently covered by the revenues. This is attributed to the cost of enzymes and the negative energy balance (**Table S5**). On the other hand, in Scenario IV, with on-site enzymes production and the use of solid hydrolyzate for energy production, the variable costs are significantly reduced (Figure 3) obtaining an acceptable gross profit margin of 60% (Table 4). However, the process is still not profitable in terms of NPV, indicating that future cash flows may not be sufficient to justify the initial investment over the long term. Considering, the impact of the capital investment the sensitivity of the financial analysis to the direct fixed cost is addressed in Section 3.4. Therefore, for an OFMSW biorefinery using enzymes to be economically viable, it should not only be energetically self-sufficient but also generate adequate revenues to cover the operating cost associated with enzymes usage and justify the increased capital investment. The integration of enzymes production on-site lowers the operating cost to an acceptable level as for lignocellulosic biorefineries [60].

In comparison with the current alternative for treating OFMSW based on anaerobic digestion and composting technologies (without heat recovery) [39], which presents a total income of 33.5 € t^−1^ of OFMSW (considering a 1.6 increase factor in the price of energy since 2019), the OFMSW biorefinery evaluated in Scenario III represents a 5-fold increase in revenues. Overall, it can be said that SSF can be integrated into OFMSW treatment plants as a supplementary tool to enhance flexibility and produce higher-value bioproducts, rather than serving as a replacement for consolidated technologies. Nevertheless, to ensure the viability of the process, the quality of the collected OFMSW must be guaranteed in the first instance.

The present study has been focused on the valorization of the solid hydrolyzate, but in literature there are plenty of examples of the valorization of the liquid fraction rich in sugars into high-value products through fermentative systems. For instance [10], proposed a profitable biorefinery scenario for the production of sophorolipids from food waste. The integration of such a process into the OFMSW biorefinery could increase its profitability. However, large capital investment and energy consumption typical of sophisticated liquid fermentation processes might represent a burden for the economic viability and further studies are required in this sense. The market value of the bioproducts and its predictability would be the key point.

### 3.4. Sensitivity analysis

**Figure 4.**
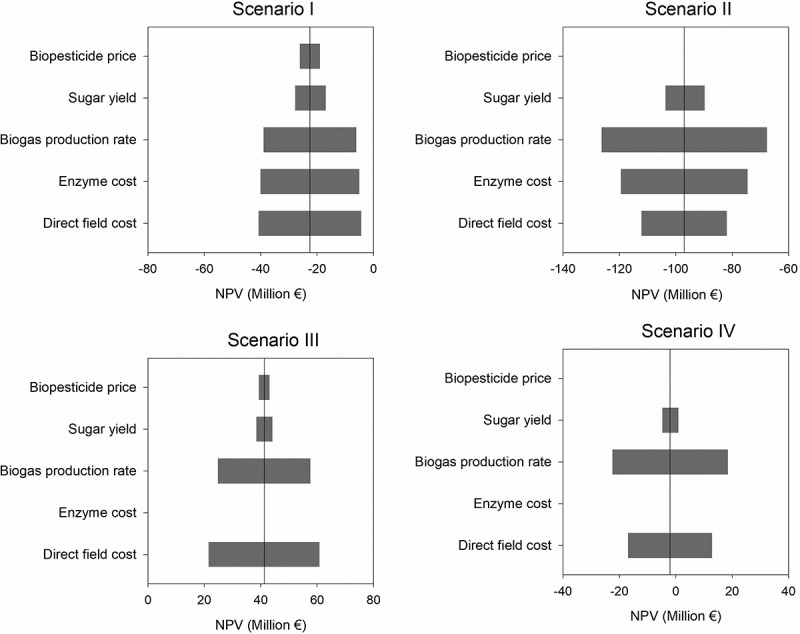
Static tornado diagram for each scenario showing the sensitivity of net present value (NPV) to the variation (±25%) in each variable while other variables are held constant. The nominal value is displayed as a vertical line.

Given the fluctuations in the global economic environment and the uncertainties in technological assumptions, it is necessary to assess the investment risk for significant parameters. The impact of fluctuations in cost parameters identified as key in Section 3.3 (i.e. enzyme cost, biopesticide price, and DFC) on the economic performance was evaluated by a sensitivity analysis. As well as, the efficiencies considered for the enzymatic hydrolysis and anaerobic digestion processes by variations in the sugar yield and biogas production rate, respectively. A tornado diagram for all scenarios is shown in Figure 4, in which each parameter is changed to ±25% of its reference value while keeping the others at the reference values. The results indicate that NPV is mostly affected by the biogas production rate, followed by the enzymes cost in Scenarios I and II and the DFC. Biogas production rate is directly related to the energy balance of the plant and, therefore, to the operating cost due to the electricity requirements. Especially, in those scenarios in which the solid hydrolyzate is used for energy production (II and IV) and biopesticides are not produced. The degree of impact from the DFC, however, depends on the capital investment specific to each scenario. For instance, in a comparison between Scenarios I and III, with a ±25% change in the DFC, the NPV is affected by about 81% in Scenario III and 16% in Scenario I. This observation can be explained by the need for additional capital investment for the SSF process for enzymes production in Scenario III, which results in a more significant impact on the NPV compared to Scenario I. Biopesticide price and sugar yield are the less influencing parameters of those studied, which can be explained considering that smaller allocation of OFMSW into the novel valorization pathway. For all the variables examined, Scenarios I and II consistently exhibited unprofitable outcomes, while Scenario III yielded profitability. Notably, Scenario IV was the most affected, indicating that with certain improvements, such as a higher biogas production rate or lower direct field costs, it may become profitable.

**Figure 5.**
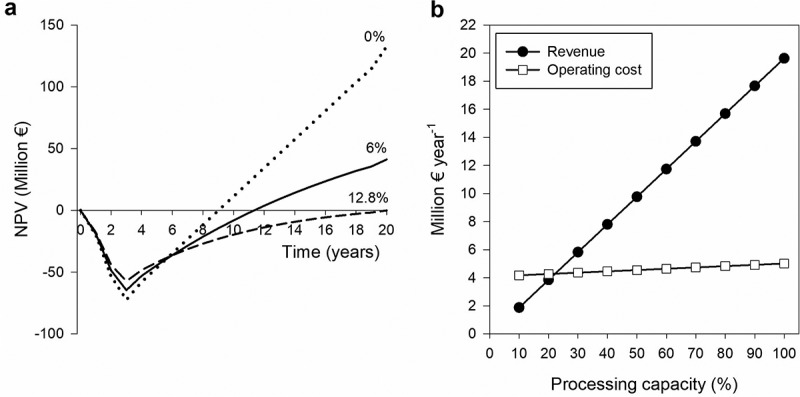
(a) Cumulative cash flow diagram at different discount rates (0%, 6% and 12.8%) for scenario III. (b) Breakeven chart of scenario III.

The profitability of Scenario III was evaluated in further detail. Cumulative cash flows at different discount rates are presented in Figure 5. The capital investment was spent during the 3-year construction time, leading to negative initial values, but then cumulative NPV increases as annual profits are generated over the lifespan of the plant. The IRR was <12.8%, indicating that the uncertainty of investment for the OFMSW biorefinery can be duplicated and still reach a break-even point by the end of the project. This value is lower in comparison with other OFMSW biorefinery configurations producing sugar syrups (15%-88%) [11] or sophorolipids (19%-36%) [10]. In both of these examples, the production of bioproducts with higher market values resulted in net present values ranging from two to seven times greater. Additionally, in other sugar syrup productions [12], the higher IRR values observed result from lower capital investment because only the sugar production pathway is considered and not an integral near zero waste approach as in this study. Finally, considering the relevance of the capital investment (Figure 4), the plant should operate at full capacity to maximize profitability. However, downtime is unavoidable due to maintenance operations, equipment failure or power cuts, and, when prolonged, it results in inefficient use of capital investment and reduces the profitability of the plant. Hence, the critical processing capacity required to reach a breakeven point was conducted. As depicted in Figure 5, the breakeven point is observed at 22%, which indicates that to generate a profit, the plant must process a minimum of 22,200 t of OFMSW waste annually.

## 4. Conclusion

In this study, four different scenarios integrating the use of enzymes in an OFMSW treatment plant using anaerobic digestion were studied based on the simulation of technically proven processes. Two novel bioproducts, biopesticide and sugar syrup, can be obtained by redirecting a portion of the incoming OFMSW. The profitability analysis of the four different scenarios assessed the origin of the enzymes and the allocation of the solid hydrolyzate. It revealed that high-value bioproducts, other than the energy produced in the anaerobic digestion, are required to justify the higher operating cost associated with the use of commercial enzymes, which represent up to 91% of the variable cost. On-site enzyme production can reduce the operating cost by 70% while increasing the capital cost by around 10%. However, further research is required to validate the enzyme production process simulated in this study. Furthermore, future research trends should focus on optimizing and scaling up low-cost production systems for obtaining enzymes from OFMSW to achieve a self-sufficient biorefinery with a closed-loop production system.

## Supplementary Material

Supplementary material.docx

## Data Availability

The data supporting the findings of this study are available within the article and its supplementary material.

## References

[1] Budzianowski WM, Postawa K. Total chain integration of sustainable biorefinery systems. Appl Energy. 2016;184:1432–18. doi: 10.1016/j.apenergy.2016.06.050

[2] Sánchez A, Artola A, Gea T, et al. A new paradigm for waste management of organic materials. Waste Manag. 2015;42:1–2. doi: 10.1016/j.wasman.2015.05.002 26026946

[3] Mayer F, Bhandari R, Gäth SA, et al. Economic and environmental life cycle assessment of organic waste treatment by means of incineration and biogasification. Is source segregation of biowaste justified in Germany? Sci Total Environ. 2020;721. doi: 10.1016/j.scitotenv.2020.137731 32208225

[4] Moreno AD, Magdalena JA, Oliva JM, et al. Sequential bioethanol and methane production from municipal solid waste: an integrated biorefinery strategy towards cost-effectiveness, process saf. Environ Prot. 2021;146:424–431. doi: 10.1016/j.psep.2020.09.022

[5] Molina-Peñate E, Artola A, Sánchez A. Organic municipal waste as feedstock for biorefineries: bioconversion technologies integration and challenges. Rev Environ Sci Bio/Technol. 2022;211(21):247–267. doi: 10.1007/S11157-021-09605-W

[6] Font X, Sánchez A. Significance of anaerobic digestion in circular bioeconomy. Biomass, Biofuels, Biochem Circ Bioeconomy-Current Dev Futur Outlook. 2021;269–289. doi: 10.1016/B978-0-12-821878-5.00020-9

[7] Fava F, Totaro G, Diels L, et al. Biowaste biorefinery in Europe: opportunities and research & development needs. New Biotechnology. 2015;32(1):100–108. doi: 10.1016/j.nbt.2013.11.003 24284045

[8] González D, Gabriel D, Sánchez A. Odors emitted from biological waste and wastewater treatment plants: a mini-review. Atmos. 2022;13(13):798. doi: 10.3390/ATMOS13050798

[9] Geissdoerfer M, Savaget P, Bocken NMP, et al. The circular economy – a new sustainability paradigm? J Clean Prod. 2017;143:757–768. doi: 10.1016/j.jclepro.2016.12.048

[10] Wang H, Tsang CW, To MH, et al. Techno-economic evaluation of a biorefinery applying food waste for sophorolipid production – a case study for Hong Kong. Bioresour Technol. 2020;303:122852. doi: 10.1016/j.biortech.2020.122852 32036326

[11] Kwan TH, Ong KL, Haque MA, et al. Biorefinery of food and beverage waste valorisation for sugar syrups production: techno-economic assessment, process Saf. Environ Prot. 2019;121:194–208. doi: 10.1016/j.psep.2018.10.018

[12] Meng F, Dornau A, Mcqueen Mason SJ, et al. Bioethanol from autoclaved municipal solid waste: assessment of environmental and financial viability under policy contexts, appl. Appl Energy. 2021;298:117118. doi: 10.1016/j.apenergy.2021.117118

[13] Ladakis D, Stylianou E, Ioannidou SM, et al. Biorefinery development, techno-economic evaluation and environmental impact analysis for the conversion of the organic fraction of municipal solid waste into succinic acid and value-added fractions. Bioresour Technol. 2022;354:127172. doi: 10.1016/j.biortech.2022.127172 35447331

[14] Demichelis F, Fiore S, Pleissner D, et al. Technical and economic assessment of food waste valorization through a biorefinery chain, renew. Sustain Energy Rev. 2018;94:38–48. doi: 10.1016/j.rser.2018.05.064

[15] Angouria-Tsorochidou E, Teigiserova DA, Thomsen M. Environmental and economic assessment of decentralized bioenergy and biorefinery networks treating urban biowaste, Resour. Conserv Recycl. 2022;176:105898. doi: 10.1016/j.resconrec.2021.105898

[16] Pleissner D, Peinemann JC. The challenges of using organic municipal solid waste as source of secondary raw materials, waste and biomass valorization. Waste Biomass Valorization. 2020;11(2):435–446. doi: 10.1007/s12649-018-0497-1

[17] Molina-Peñate E, Del Carmen Vargas-García M, Artola A, et al. Filling in the gaps in biowaste biorefineries: the use of the solid residue after enzymatic hydrolysis for the production of biopesticides through solid-state fermentation. Waste Manag. 2023;161:92–103. doi: 10.1016/j.wasman.2023.02.029 36871406

[18] Mejias L, Estrada M, Barrena R, et al. A novel two-stage aeration strategy for *Bacillus thuringiensis* biopesticide production from biowaste digestate through solid-state fermentation. Biochem Eng J. 2020;161:107644. doi: 10.1016/j.bej.2020.107644

[19] Rodríguez P, Cerda A, Font X, et al. Valorisation of biowaste digestate through solid state fermentation to produce biopesticides from *Bacillus thuringiensis*. Waste Manag. 2019;93:63–71. doi: 10.1016/j.wasman.2019.05.026 31235058

[20] Sala A, Barrena R, Artola A, et al. Current developments in the production of fungal biological control agents by solid-state fermentation using organic solid waste. Crit Rev Environ Sci Technol. 2019;49(8):655–694. doi: 10.1080/10643389.2018.1557497

[21] Thakur N, Kaur S, Tomar P, et al. Microbial biopesticides: Current status and advancement for sustainable agriculture and environment, New Futur. Dev Microb Biotechnol Bioeng Trends Microb Biotechnol Sustain Agric Biomed Syst Divers Funct Perspec. 2020;243–282. doi: 10.1016/b978-0-12-820526-6.00016-6

[22] Biopesticides market size & share analysis - industry research report - growth trends. [cited 2023 Oct 12]. https://www.mordorintelligence.com/industry-reports/global-biopesticides-market-industry

[23] Bravo A, Likitvivatanavong S, Gill SS, et al. *Bacillus thuringiensis*: a story of a successful bioinsecticide. Insect Biochem Mol Biol. 2011;41(7):423–431. doi: 10.1016/j.ibmb.2011.02.006 21376122 PMC3689885

[24] Le Pera A, Sellaro M, Pellegrino C, et al. Improved full-scale anaerobic digestion of food waste: a core technology in the biorefinery approach. Bioresour Technol Reports. 2022;19:101126. doi: 10.1016/j.biteb.2022.101126

[25] Guo H, Chang Y, Lee DJ. Enzymatic saccharification of lignocellulosic biorefinery: research focuses. Bioresour Technol. 2018;252:198–215. doi: 10.1016/j.biortech.2017.12.062 29329774

[26] Biz A, Finkler ATJ, Pitol LO, et al. Production of pectinases by solid-state fermentation of a mixture of citrus waste and sugarcane bagasse in a pilot-scale packed-bed bioreactor. Biochem Eng J. 2016;111:54–62. doi: 10.1016/j.bej.2016.03.007

[27] Cerda A, Mejías L, Gea T, et al. Cellulase and xylanase production at pilot scale by solid-state fermentation from coffee husk using specialized consortia: the consistency of the process and the microbial communities involved. Bioresour Technol. 2017;243:1059–1068. doi: 10.1016/j.biortech.2017.07.076 28764108

[28] Llimós J, Martínez-Avila O, Marti E, et al. Brewer’s spent grain biotransformation to produce lignocellulolytic enzymes and polyhydroxyalkanoates in a two-stage valorization scheme, biomass convers. Biomass Conv Bioref. 2022;12(9):3921–3932. doi: 10.1007/S13399-020-00918-4/

[29] Pranay K, Padmadeo SR, Prasad B. Production of amylase from *Bacillus subtilis sp*. strain KR1 under solid state fermentation on different agrowastes. Biocatal Agric Biotechnol. 2019;21:101300. doi: 10.1016/j.bcab.2019.101300

[30] Residuos municipales. Agència de Residus de Catalunya. [cited 2023 Oct 6]. https://residus.gencat.cat/es/ambits_dactuacio/recollida_selectiva/residus_municipals/

[31] Molina-Peñate E, Sánchez A, Artola A. Enzymatic hydrolysis of the organic fraction of municipal solid waste: optimization and valorization of the solid fraction for *Bacillus thuringiensis* biopesticide production through solid-state fermentation. Waste Manag. 2022;137:304–311. doi: 10.1016/j.wasman.2021.11.014 34823137

[32] Molina-Peñate E, Arenòs N, Sánchez A, et al. *Bacillus thuringiensis* production through solid-state fermentation using organic fraction of municipal solid waste (OFMSW) enzymatic hydrolysate. Waste Biomass Valorization. 2023;14(5):1433–1445. doi: 10.1007/s12649-022-01978-5

[33] López-Gómez JP, Latorre-Sánchez M, Unger P, et al. Assessing the organic fraction of municipal solid wastes for the production of lactic acid. Biochem Eng J. 2019;150. doi: 10.1016/j.bej.2019.107251

[34] Kuo PC, Yu J. Process simulation and techno-economic analysis for production of industrial sugars from lignocellulosic biomass, Ind. Crops Prod. 2020;155:112783. doi: 10.1016/j.indcrop.2020.112783

[35] Manjunatha GS, Chavan D, Lakshmikanthan P, et al. Specific heat and thermal conductivity of municipal solid waste and its effect on landfill fires. Waste Manag. 2020;116:120–130. doi: 10.1016/j.wasman.2020.07.033 32795643

[36] Violidakis I, Drosatos P, Nikolopoulos N. Critical review of current industrial scale lignite drying technologies, low-rank coals power gener. Fuel Chem Prod. 2017;41–71. doi: 10.1016/B978-0-08-100895-9.00003-6

[37] Puyuelo B, Gea T, Sánchez A. A new control strategy for the composting process based on the oxygen uptake rate. Chem Eng J. 2010;165:161–169. doi: 10.1016/j.cej.2010.09.011

[38] Meyer H-P, Minas W, Schmidhalter D. Industrial-scale fermentation, Ind. Biotechnol. 2017;1–53. doi: 10.1002/9783527807833.ch1

[39] Abad V, Avila R, Vicent T, et al. Promoting circular economy in the surroundings of an organic fraction of municipal solid waste anaerobic digestion treatment plant: biogas production impact and economic factors. Bioresour Technol. 2019;283:10–17. doi: 10.1016/j.biortech.2019.03.064 30897388

[40] Khoshnevisan B, Tsapekos P, Alvarado-Morales M, et al. Life cycle assessment of different strategies for energy and nutrient recovery from source sorted organic fraction of household waste. J Clean Prod. 2018;180:360–374. doi: 10.1016/j.jclepro.2018.01.198

[41] Tampio E, Marttinen S, Rintala J. Liquid fertilizer products from anaerobic digestion of food waste: mass, nutrient and energy balance of four digestate liquid treatment systems. J Clean Prod. 2016;125:22–32. doi: 10.1016/j.jclepro.2016.03.127

[42] Rajendran K, Kankanala HR, Martinsson R, et al. Uncertainty over techno-economic potentials of biogas from municipal solid waste (MSW): a case study on an industrial process, appl. Appl Energy. 2014;125:84–92. doi: 10.1016/j.apenergy.2014.03.041

[43] Lantz M. The economic performance of combined heat and power from biogas produced from manure in Sweden – a comparison of different CHP technologies, appl. Appl Energy. 2012;98:502–511. doi: 10.1016/j.apenergy.2012.04.015

[44] Seruga P, Krzywonos M, Seruga A, et al. Anaerobic digestion performance: separate collected vs. mechanical segregated organic fractions of municipal solid waste as feedstock. Energies. 2020;13(15):3768. doi: 10.3390/en13153768

[45] Rapport JL, Zhang R, Jenkins BM, et al. Modeling the performance of the anaerobic phased solids digester system for biogas energy production. Biomass Bioenergy. 2011;35(3):1263–1272. doi: 10.1016/j.biombioe.2010.12.021

[46] Liu G, Zhang J, Bao J. Cost evaluation of cellulase enzyme for industrial-scale cellulosic ethanol production based on rigorous Aspen Plus modeling. Bioprocess Biosyst Eng. 2016;39(1):133–140. doi: 10.1007/s00449-015-1497-1 26541585

[47] Stylianou E, Pateraki C, Ladakis D, et al. Evaluation of organic fractions of municipal solid waste as renewable feedstock for succinic acid production. Biotechnol Biofuels. 2020;13(1):1–16. doi: 10.1186/s13068-020-01708-w 32322302 PMC7160979

[48] Abdullah J, Greetham D. Optimizing cellulase production from municipal solid waste (MSW) using solid state fermentation (SSF). Artic J Fundam Renew Energy Appl. 2016 3;6(3). doi: 10.4172/2090-4541.1000206

[49] Towler G, Sinnott R. Chemical engineering design: principles, practice and economics of plant and process design. Oxford: Butterworth-Heinemann; 2021.

[50] Ulrich GD. A Guide to chemical engineering process design and economics. New York (NY): Wiley; 1984.

[51] Woods DR. Rules of thumb in engineering practice, rules thumb eng. Pract. 2007;1–458. doi: 10.1002/9783527611119

[52] Preus de l’energia. Institut Català d’Energia. [cited 2023 Oct 11]. https://icaen.gencat.cat/ca/energia/preus/

[53] Precio por municipios y evolución. Agencia Catalana del Agua. [cited 2023 Oct 11]. https://aca.gencat.cat/es/laca/observatori-del-preu-de-laigua/Preu-per-municipis-i-evolucio/index.html

[54] Zhang C, Bozileva E, van de Klis F, et al. Integration of galacturonic acid extraction with alkaline protein extraction from green tea leaf residue, Ind. Crops Prod. 2016;89:95–102. doi: 10.1016/j.indcrop.2016.04.074

[55] ¿Qué es la FORM?. Agència de Residus de Catalunya. [cited 2023 Oct 11]. https://residus.gencat.cat/es/ambits_dactuacio/recollidaselectiva/residusmunicipals/materiaorganicaformfv/queeslaform/

[56] Chen X, Shekiro J, Pschorn T, et al. Techno-economic analysis of the deacetylation and disk refining process: characterizing the effect of refining energy and enzyme usage on minimum sugar selling price and minimum ethanol selling price. Biotechnol Biofuels. 2015;8(1):1–13. doi: 10.1186/S13068-015-0358-0 26516346 PMC4625976

[57] Andreola C, González-Camejo J, Tambone F, et al. Techno-economic assessment of biorefinery scenarios based on mollusc and fish residuals. Waste Manag. 2023;166:294–304. doi: 10.1016/j.wasman.2023.05.014 37207590

[58] Panigrahi S, Dubey BK. A critical review on operating parameters and strategies to improve the biogas yield from anaerobic digestion of organic fraction of municipal solid waste, renew. Renewable Energy. 2019;143:779–797. doi: 10.1016/j.renene.2019.05.040

[59] Kendir Çakmak E, Ugurlu A. Enhanced biogas production of red microalgae via enzymatic pretreatment and preliminary economic assessment. Algal Res. 2020;50:101979. doi: 10.1016/j.algal.2020.101979

[60] Johnson E. Integrated enzyme production lowers the cost of cellulosic ethanol. Biofuels, Bioprod Biorefining. 2016;10(2):164–174. doi: 10.1002/bbb.1634

