# Supplementary material for: Exploring biorefinery alternatives for biowaste valorization: a techno-economic assessment of enzymatic hydrolysis coupled with anaerobic digestion or solid-state fermentation for high-value bioproducts
Source: Bioengineered. 2024 Jan 24;15(1):2307668. doi: 10.1080/21655979.2024.2307668 (PMC10810166; doi:10.1080/21655979.2024.2307668)
Supplement: Supplementary material.docx [file KBIE_A_2307668_SM0342.docx]

Supplementary material for

# Techno-economical assessment of different biorefinery scenarios for biowaste valorization based on enzymatic hydrolysis, anaerobic digestion and solid-state fermentation

Esther Molina-Peñate ^a^, Adriana Artola ^a^, Antoni Sánchez ^a,^^[[1]](#footnote-1)^

^a^ *GICOM Research Group, Department of Chemical, Biological and Environmental Engineering, School of Engineering, Edifici Q, Universitat Autònoma de Barcelona, 08193, Bellaterra, Barcelona, Spain*

ORCID numbers: 0000-0001-9590-4739, 0000-0002-0524-2119, 0000-0003-4254-8528

**1. Mass balances**

**Table S1**. Major design parameters of the OFMSW biorefinery.

| **Unit procedure** | **Parameter** | | **Value** | | | **Reference** | | |
| --- | --- | --- | --- | --- | --- | --- | --- | --- |
| *Pretreatment* |  | | | |  | |  | |
| Shredder | Processing capacity (t h^-1^)  Electricity consumption (kW) | | | | 15  110 | | HAMMEL VB 450E ® | |
| Autoclave | Processing capacity (t batch^-1^)  Batch duration (h)  Electricity consumption (MJ t^-1^)  Natural gas consumption (MJ t^-1^)  Water consumption (L t^-1^) | | | | 20  4  43  274  245 | | [1] | |
| *Enzymatic hydrolysis* |  | | | |  | |  | |
| Hydrolysis tank | Batch duration (h)  Temperature (ºC)  Solid load (%)  Heat capacity (kJ kg^-1^ ºC^-1^) | | | | 48  50  10  3.3 | | [2,3] | |
| Centrifuge | Processing capacity (t h^-1^)  Solid recovery from input (%)  Electricity consumption (kW) | | | | 15  26  146 | | CDE group® | |
| Dryer | Processing capacity (t water h^-1^)  Electricity consumption (MJ kg^-1^ water) | | | | 10  3 | | [4] | |
| *Anaerobic digestion* |  | | | |  | |  | |
| Bioreactor | Solid load (%)  Loading rate (kg VS m^-3^ day^-1^)  Hydraulic retention time (day) | | | | 15  3  16 | | [5] | |
| Combustion engine | Engine power (kW)  Electricity consumption (% generated) | | | | 2000  10 | | [6] | |
| Centrifuge | Processing capacity (t h^-1^)  Solid recovery from input (%)  Electricity consumption (kW) | | | | 15  20  146 | | CDE group®  [7] | |
| Hygienization tank | Batch duration (h)  Temperature (ºC)  Heat capacity (kJ kg^-1^ ºC^-1^) | | | | 6  75  4.18 | |  | |
| *SSF for biopesticide production* |  |  | |  | | | |  |
| Seed bioreactor | Batch duration (h)  Electricity consumption (kW m^-3^) | | | | 48  4 | |  | |
| SSF bioreactor | Processing capacity (t batch^-1^)  Batch duration (day)  Air consumption (m^3^ day^-1^ t^-1^)  Compressor capacity (m^3^ h^-1^)  Electricity consumption (kWh t^-1^) | | | | 40  6  250  417  27.5 | | [8,9] | |
| Sieve | Processing capacity (t h^-1^)  Electricity consumption (kW) | | | | 30  40 | | T4 Terra Select ® | |
| *SSF for enzymes production* |  | | | |  | |  | |
| Seed bioreactor | Batch duration (h)  Electricity consumption (kW m^-3^) | | | | 120  4 | |  | |
| SSF bioreactor | Processing capacity (t batch^-1^)  Batch duration (day)  Air consumption (m^3^ day^-1^ t^-1^)  Compressor capacity (m^3^ h^-1^)  Electricity consumption (kWh t^-1^) | | | | 40  5  250  313  27.5 | |  | |
| Sieve | Processing capacity (t h^-1^)  Electricity consumption (kW) | | | | 10  40 | | T4 Terra Select ® | |

The volume of biogas produced during the anaerobic digestion is estimated based on the inlet substrate at a conversion rate of 151 m^3^ t^-1^ of wet OFMSW [10] and it can be converted to mass of biogas following the equation:

*m = (V × MC × d_m_ /1000) + (V × CC × d_c_ /1000)*

where *m* is the mass of biogas (t year^-1^), *V* is the volume of biogas (m^3^ year^-1^), *MC* is the assumed methane content (64%), *d_m_* is the density of methane (0.72 kg m^-3^), *CC* is the assumed carbon dioxide content (36%), *d_c_* is the density of carbon dioxide (1.96 kg m^-3^).

The biogas mass is subsequently used to calculate the digestate mass by subtracting it from the substrate mass.

**Table S2**. Characteristics of the principal solid materials of the biorefinery: OFMSW, solid digestate and solid enzymatic hydrolysate [8].

| **Parameter** | **OFMSW** | **Enzymatic hydrolysate** | **Solid digestate** |
| --- | --- | --- | --- |
| Moisture content (%) | 76.4 ± 1.1 | 77.4 ± 2.4 | 77.2 ± 0.4 |
| Dry matter (%) | 23.6 ± 1.1 | 22.6 ± 2.4 | 22.8 ± 0.4 |
| Organic matter (%*) | 89.7 ± 0.7 | 86.9 ± 1.7 | 72.3 ± 6.3 |
| Reducing sugars (%*) | 16.6 ± 0.9 | 12.6 ± 1.7 | - |
| pH | 5.6 ± 0.1 | 5.3 ± 0.1 | 8.5 ± 0.2 |
| Conductivity (mS/cm) | 2.2 ± 0.1 | 2.6 ± 0.1 | 2.6 ± 0.1 |

*dry basis

**2. Energy balances**

The amount of energy needed for heating purposes, such as the enzymatic hydrolysis (50ºC), the anaerobic digestion (40ºC) and the hygienization (75ºC) is estimated according to the equation:

*ΔE = c × m × ΔT*

where *ΔE* is the energy needed for heating the different mixtures, *c* is the specific heat capacity (kJ kg^-1^ ºC^-1^), *m* is the mass of the mixture (kg) and *ΔT* is the change of the temperature from ambient temperature (20ºC) to the final.

For the energy (electrical and thermal) generated in the anaerobic digestion unit, the following formula was used:

*Energy yield (kWh t^-1^) = biogas yield (m^3^ t^-1^) × methane content (%) × energy potential (kWh m^-3^ CH_4_) × CHP unit efficiency*

A conversion factor of 10kWh m^-3^ methane was used for the energy potential and the CHP unit efficiency was 38% for electricity and 48% for heat [7]. The energy consumption of the CHP unit was set to a 10% of all the energy generated and the heat losses in the biorefinery were assumed to be a 20% of the heat demand, which is a conservative estimate as lower values have been reported [7].

**Table S3**. Detailed energy consumption of each equipment of the biorefinery for each scenario.

| **Item** | | **Quantity (MWh year-^1^)** | | | | | | |
| --- | --- | --- | --- | --- | --- | --- | --- | --- |
|  |  | **Scenario I** | | **Scenario II** | | **Scenario III** | | **Scenario IV** |
| **Equipment purchase cost** | |  | |  | |  | |  |
| *Pretreatment* | |  | |  | |  | |  |
| Shredder | | 696 | | 696 | | 696 | | 696 |
| Autoclave | | 2,642 | | 2,642 | | 2,642 | | 2,642 |
| *Enzymatic hydrolysis* | |  | |  | |  | |  |
| Hydrolysis tank | | 2,056 | | 2,056 | | 1,094 | | 1,094 |
| Centrifuge | | 738 | | 738 | | 389 | | 389 |
| Dryer | | 42,105 | | 42,105 | | 22,153 | | 22,153 |
| *Anaerobic digestion* | |  | |  | |  | |  |
| Bioreactor | | 4,841 | | 6,205 | | 4,841 | | 5,559 |
| Combustion engine | | 5,168 | | 6,625 | | 5,168 | | 5,935 |
| Centrifuge | | 970 | | 1,243 | | 970 | | 1,114 |
| Hygienization tank | | 1,273 | | 1,632 | | 1,273 | | 1,462 |
| *SSF for biopesticide production* |  | |  | |  | |  | |
| Seed bioreactor | | 320 | | 0 | | 320 | | 0 |
| SSF bioreactor | | 1,418 | | 0 | | 1,084 | | 0 |
| Sieve | | 70 | | 0 | | 54 | | 0 |
| *SSF for enzymes production* | |  | |  | |  | |  |
| Seed bioreactor | | 0 | | 0 | | 128 | | 128 |
| SSF bioreactor | | 0 | | 0 | | 536 | | 536 |
| Sieve | | 0 | | 0 | | 78 | | 78 |
| **Total energy consumption** | | **62,297** | | **63,942** | | **41,425** | | **41,784** |
|  | |  | |  | |  | |  |

**Figure S1**. Energy consumption distributed per equipment of the biorefinery for each scenario.


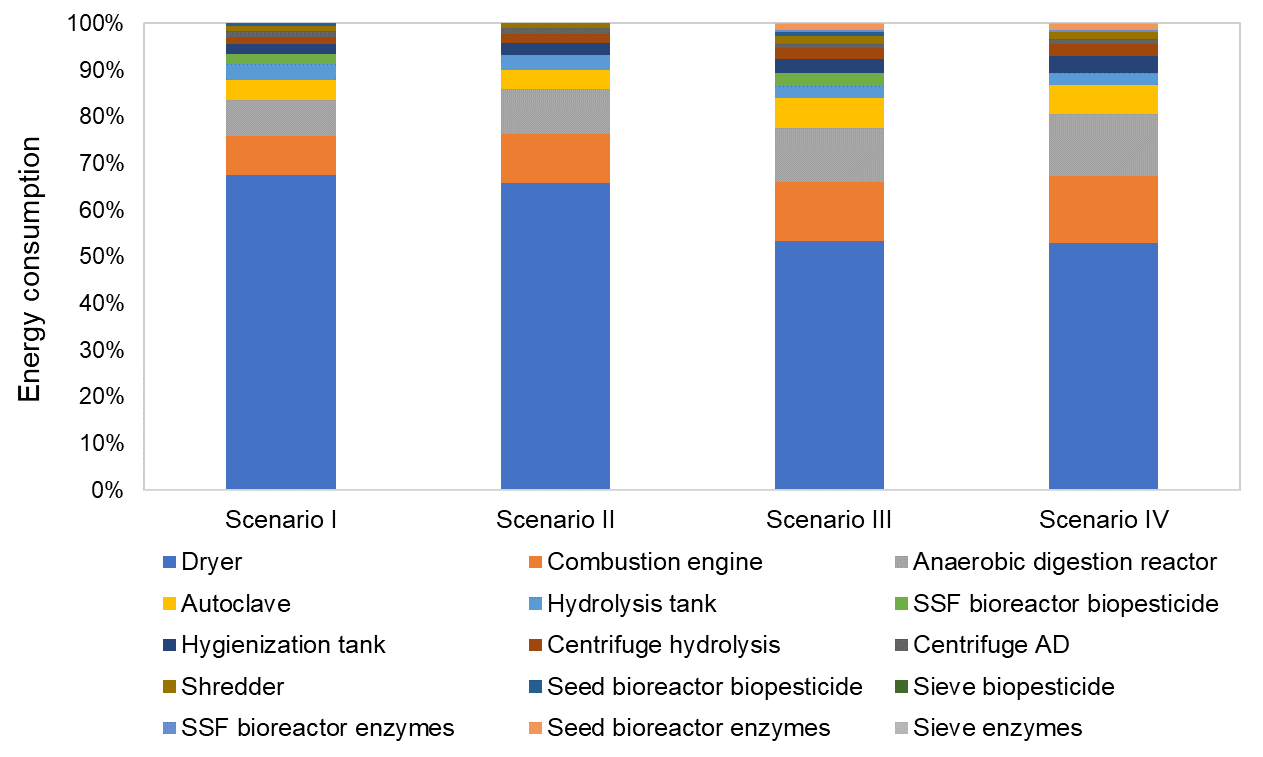


**3. Economic assessment**

The equipment purchase costs have been estimated by a correlation between a base cost and specific equipment cost-dependent parameter, according to the formula [11]:

$${Cost}_{2}={Cost}_{ref}\times({\frac{{Parameter}_{2}}{{Parameter}_{ref}})}^{n}$$

Carbon steel has been considered as the equipment material.

The economic analysis has been done in €, the conversion of other currencies applied have been: 1.09$ and 0.87GBP.

**Table S4**. Detailed capital investment (€) of the biorefinery for each scenario.

| **Item** | | **Quantity / Cost (€)** | | | | | | |
| --- | --- | --- | --- | --- | --- | --- | --- | --- |
|  |  | **Scenario I** | | **Scenario II** | | **Scenario III** | | **Scenario IV** |
| **Equipment purchase cost** | |  | |  | |  | |  |
| *Pretreatment* | |  | |  | |  | |  |
| Shredder (15 t h^-1^) | | 1 / 139,000 | | 1 / 139,000 | | 1 / 139,000 | | 1 / 139,000 |
| Autoclave (40 m^3^) | | 1 / 340,400 | | 1 / 340,400 | | 1 / 340,400 | | 1 / 340,400 |
| *Enzymatic hydrolysis* | |  | |  | |  | |  |
| Hydrolysis tank (400 m^3^) | | 2 / 157,892 | | 2 / 157,892 | | 2 / 157,892 | | 1 / 78,946 |
| Centrifuge (15 t h^-1^) | | 1 / 593,400 | | 1 / 593,400 | | 1 / 593,400 | | 1 / 593,400 |
| Dryer (10 t water h^-1^) | | 1 / 1,082,840 | | 1 / 1,082,840 | | 1 / 1,082,840 | | 1 / 1,082,840 |
| *Anaerobic digestion* | |  | |  | |  | |  |
| Bioreactor (3000 m^3^) | | 5 / 1,380,000 | | 7 / 1,932,000 | | 5 / 1,380,000 | | 6 / 1,656,000 |
| Combustion engine (2000 kW) | | 1 / 678,000 | | 1 / 678,000 | | 1 / 678,000 | | 1 / 678,000 |
| Centrifuge (15 t h^-1^) | | 1 / 593,400 | | 1 / 593,400 | | 1 / 593,400 | | 1 / 593,400 |
| Hygienization tank (100 m^3^) | | 1 / 78,946 | | 1 / 78,946 | | 1 / 78,946 | | 1 / 78,946 |
| *SSF for biopesticide production* |  | |  | |  | |  | |
| Seed bioreactor (10 m^3^) | | 2 / 344,632 | | - | | 2 / 344,632 | | - |
| SSF bioreactor (50 m^3^) | | 6 / 2,100,000 | | - | | 5 / 1,750,000 | | - |
| Sieve (30 t h^-1^) | | 1 / 41,800 | | - | | 1 / 41,800 | | - |
| *SSF for enzymes production* | |  | |  | |  | |  |
| Seed bioreactor (1 m^3^) | | - | | - | | 5 / 216,430 | | 5 / 216,430 |
| SSF bioreactor (50 m^3^) | | - | | - | | 2 / 700,000 | | 2 / 700,000 |
| Sieve (10 t h^-1^) | | - | | - | | 1 / 14,000 | | 1 / 14,000 |
| Total | | 7,530,310 | | 5,595,878 | | 8,110,740 | | 6,171,362 |
|  | |  | |  | |  | |  |
| Installation cost | | 24,096,993 | | 17,906,811 | | 25,954,369 | | 19,748,359 |
| Off-site cost | | 12,650,921 | | 9,401,076 | | 13,626,044 | | 10,367,889 |
| Engineering cost | | 11,069,556 | | 8,225,941 | | 11,922,788 | | 9,071,902 |
| Contingency cost | | 6,641,734 | | 4,935,565 | | 7,153,673 | | 5,443,141 |
| Working capital | | 6,641,734 | | 4,935,565 | | 7,153,673 | | 5,443,141 |
| **Total capital investment** | | **68,631,249** | | **51,000,835** | | **73,921,288** | | **56,245,795** |

*In brackets the specification for the cost estimation of each equipment is indicated.

**Table S5**. Detailed operating costs (€ year^-1^) of the biorefinery for each scenario.

| **Item** | **Cost (€ year^-1^)** | | | |
| --- | --- | --- | --- | --- |
|  | **Scenario I** | **Scenario II** | **Scenario III** | **Scenario IV** |
| **Variable operating cost (VOC)** |  |  |  |  |
| *Utilities* |  |  |  |  |
| Electricity | 2,348,891 | - | - | - |
| Water | 342,756 | 365,111 | 169,560 | 179,840 |
| *Raw materials* |  |  |  |  |
| Commercial enzymes | 9,936,000 | 9,936,000 | - | - |
| Rich media | 257,783 | - | 200,929 | 3,900 |
| *Other* |  |  |  |  |
| Wastewater treatment | 494,000 | 494,000 | 494,000 | 494,000 |
| Laboratory & analysis | 72,000 | 72,000 | 72,000 | 72,000 |
| Total | 13,451,429 | 10,867,111 | 932,589 | 749,740 |
| **Fixed operating cost (FOC)** |  |  |  |  |
| Labor | 1,572,000 | 1,572,000 | 1,572,000 | 1,572,000 |
| Maintenance | 1,897,638 | 1,410,161 | 2,043,907 | 1,555,183 |
| Insurance | 433,927 | 322,457 | 467,373 | 355,619 |
| Total | 3,903,565 | 3,304,618 | 4,083,280 | 3,483,802 |
| **Total operating cost** | **17,354,994** | **14,171,729** | **5,015,869** | **4,232,542** |

**Figure S2**. Cumulative cash flow diagrams for each scenario at a 6% discount rate.


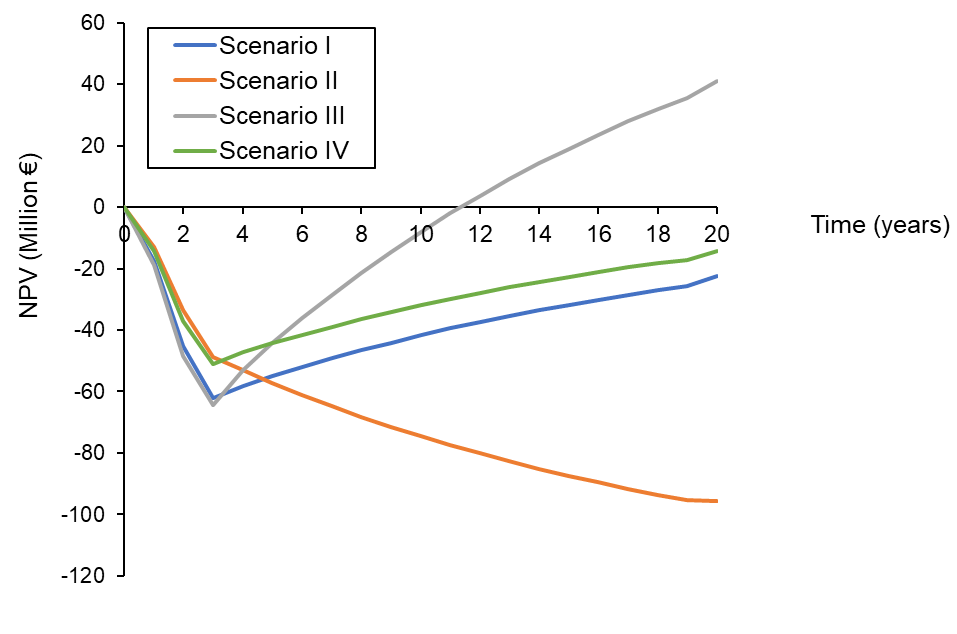


**Table S6**. Parameters values for sensitivity analysis of the biorefinery.

| **Parameter** | **-25%** | **Base case** | **+25%** |
| --- | --- | --- | --- |
| Enzyme cost (€ kg^-1^) | 10.35 | 13.8 | 17.25 |
| Price of biopesticide (€ kg^-1^) | 0.274 | 0.365 | 0.456 |
| Direct field cost (Million €) | 23.72 | 31.63 | 39.53 |
| Biogas production rate (m^3^ t^-1^ OFMSW) | 113.3 | 151 | 188.8 |
| Sugar yield (g L^-1^) | 38 | 50 | 63 |

**References**

[1] F. Meng, A. Dornau, S.J. Mcqueen Mason, G.H. Thomas, A. Conradie, J. McKechnie, Bioethanol from autoclaved municipal solid waste: Assessment of environmental and financial viability under policy contexts, Appl. Energy. 298 (2021) 117118. https://doi.org/10.1016/J.APENERGY.2021.117118.

[2] E. Molina-Peñate, A. Sánchez, A. Artola, Enzymatic hydrolysis of the organic fraction of municipal solid waste: Optimization and valorization of the solid fraction for Bacillus thuringiensis biopesticide production through solid-state fermentation, Waste Manag. 137 (2022) 304–311. https://doi.org/10.1016/J.WASMAN.2021.11.014.

[3] G.S. Manjunatha, D. Chavan, P. Lakshmikanthan, L. Singh, S. Kumar, R. Kumar, Specific heat and thermal conductivity of municipal solid waste and its effect on landfill fires, Waste Manag. 116 (2020) 120–130. https://doi.org/10.1016/J.WASMAN.2020.07.033.

[4] I. Violidakis, P. Drosatos, N. Nikolopoulos, Critical review of current industrial scale lignite drying technologies, Low-Rank Coals Power Gener. Fuel Chem. Prod. (2017) 41–71. https://doi.org/10.1016/B978-0-08-100895-9.00003-6.

[5] V. Abad, R. Avila, T. Vicent, X. Font, Promoting circular economy in the surroundings of an organic fraction of municipal solid waste anaerobic digestion treatment plant: Biogas production impact and economic factors, Bioresour. Technol. 283 (2019) 10–17. https://doi.org/10.1016/J.BIORTECH.2019.03.064.

[6] M. Lantz, The economic performance of combined heat and power from biogas produced from manure in Sweden – A comparison of different CHP technologies, Appl. Energy. 98 (2012) 502–511. https://doi.org/10.1016/J.APENERGY.2012.04.015.

[7] E. Tampio, S. Marttinen, J. Rintala, Liquid fertilizer products from anaerobic digestion of food waste: mass, nutrient and energy balance of four digestate liquid treatment systems, J. Clean. Prod. 125 (2016) 22–32. https://doi.org/10.1016/J.JCLEPRO.2016.03.127.

[8] E. Molina-Peñate, M. del Carmen Vargas-García, A. Artola, A. Sánchez, Filling in the gaps in biowaste biorefineries: The use of the solid residue after enzymatic hydrolysis for the production of biopesticides through solid-state fermentation, Waste Manag. 161 (2023) 92–103. https://doi.org/10.1016/J.WASMAN.2023.02.029.

[9] B. Puyuelo, T. Gea, A. Sánchez, A new control strategy for the composting process based on the oxygen uptake rate, Chem. Eng. J. 165 (2010) 161–169. https://doi.org/10.1016/J.CEJ.2010.09.011.

[10] B. Khoshnevisan, P. Tsapekos, M. Alvarado-Morales, S. Rafiee, M. Tabatabaei, I. Angelidaki, Life cycle assessment of different strategies for energy and nutrient recovery from source sorted organic fraction of household waste, J. Clean. Prod. 180 (2018) 360–374. https://doi.org/10.1016/J.JCLEPRO.2018.01.198.

[11] D.R. Woods, Rules of Thumb in Engineering Practice, Rules Thumb Eng. Pract. (2007) 1–458. https://doi.org/10.1002/9783527611119.

1. **Corresponding author**: Antoni Sánchez ([antoni.sanchez@uab.cat](mailto:antoni.sanchez@uab.cat)) [↑](#footnote-ref-1)
